# Supplementary material for: Exploratory Assessment of Proteomic Network Changes in Cerebrospinal Fluid of Mild Cognitive Impairment Patients: A Pilot Study
Source: Biomolecules. 2023 Jul 8;13(7):1094. doi: 10.3390/biom13071094 (PMC10377001; doi:10.3390/biom13071094)

Supplementary Material

Methods

Multivariate Association Analysis

To estimate the statistical significance of each factor as a main effect and interacting with other factors, a set of 4 models of increasing complexity were compared with a sequential F test. This test is a measure of the marginal reduction in the error sum of squares when additional predictors are added to the model. The four models are:

$$Y \sim \beta 1 + \varepsilon \quad (1)$$

$$Y \sim \beta 1 + \beta 2 \,{*}\text{main\_effect} + \varepsilon \quad (2)$$

$$Y \sim \beta 1 + \beta 2{*}\text{age} + \beta 3{*}\text{APOE} + \beta 4{*}\text{cog} + \beta 5{*}\text{sex} + \varepsilon \quad (3)$$

$$Y \sim \beta 1 + \beta 2{*}\text{age} + \beta 3{*}\text{APOE} + \beta 4{*}\text{cog} + + \beta 5{*}\text{sex} + \beta 6{*}\text{main\_effect}{*}\text{cog} + \varepsilon \quad (4)$$

Model (1) provides an estimate of the variance around the grand mean. This model is used only to estimate variance for comparison to the other models. Model (2) introduces the main effects to be tested (e.g., main_effect will be age, sex, APOE or diagnosis (cog)). APOE is coded for presence or absence of at least one ε4 allele. Diagnosis is coded for MCI or normal cognition. A comparison of model (1) and (2) estimates the effect (beta coefficient) for each main factor in turn and the sequential F test tests the marginal reduction in the sum of squares for adding each factor in turn (e.g., diagnosis, age, sex, APOE ε4 presence/absence). The comparison of model (3) and (2) tests the marginal reduction in the sum of squares by adding the remaining main effects to the model (e.g., adding age, sex, and APOE ε4 presence/absence to a model that only includes diagnosis) while the comparison of model (4) and (3) introduces an interaction term of a specific main effect with diagnosis, allowing for that interaction to be tested. FDR-adjusted p-values are reported for the pair-wise model comparisons. The beta coefficient and standard error for each factor (β2 in model 2) is reported to evaluate the effect size for each variable of interest.

The univariate Welch's two-sample t-test, which is the default analysis platform for the OLINK software, identified differential expression of the proteins by diagnosis and dichotomized age and provided mean group-differences for the factors of interest and FDR-corrected p-values.

In comparison, multivariate analysis allowed estimation of the effect of each factor on protein concentration when diagnosis, age, sex and APOE genotype were included as covariates. The most complex model allowed for an interaction between age and diagnosis. While the multivariate analysis identified more proteins as significant based on FDR-corrected p values, the differences in the hypotheses tested and caveats of interpreting p values must be considered. The t-test is a univariate test of differences between the means for the group comparisons (diagnosis or dichotomized age). The multivariate analyses tests whether each factor, individually, with the other covariates in the model or allowing for and interaction between diagnosis and age shows a significant effect in contrast to a more parsimonious model. The effect sizes for the factors in each model are also reported for interpretation.

The analytical results from the two approaches are complementary and can be used to plan future experiments. The supplemental tables allow analysis of individual protein concentrations stratified by each of the covariates and construction of protein sets for gene set enrichment analysis or over-enrichment analysis for the different statistical models.

Statistical Power Calculations

In this study, we conducted power calculations to determine the statistical power of our assay hits for CHI3L1 and SCRN1. We calculated effect sizes for CHI3L1 and SCRN1, and the results showed that both proteins had good statistical power with effect sizes of 0.79 and 1.14, respectively. We also calculated the power at alpha levels of 0.05 and 0.001, which were 0.95 and 0.59 for CHI3L1, and 0.99 and 0.96 for SCRN1, respectively. Following standard practice for proteomic studies we used a false discovery rate (FDR) correction to account for multiple comparisons. The diagrams below show the power calculations of CHI3L1.

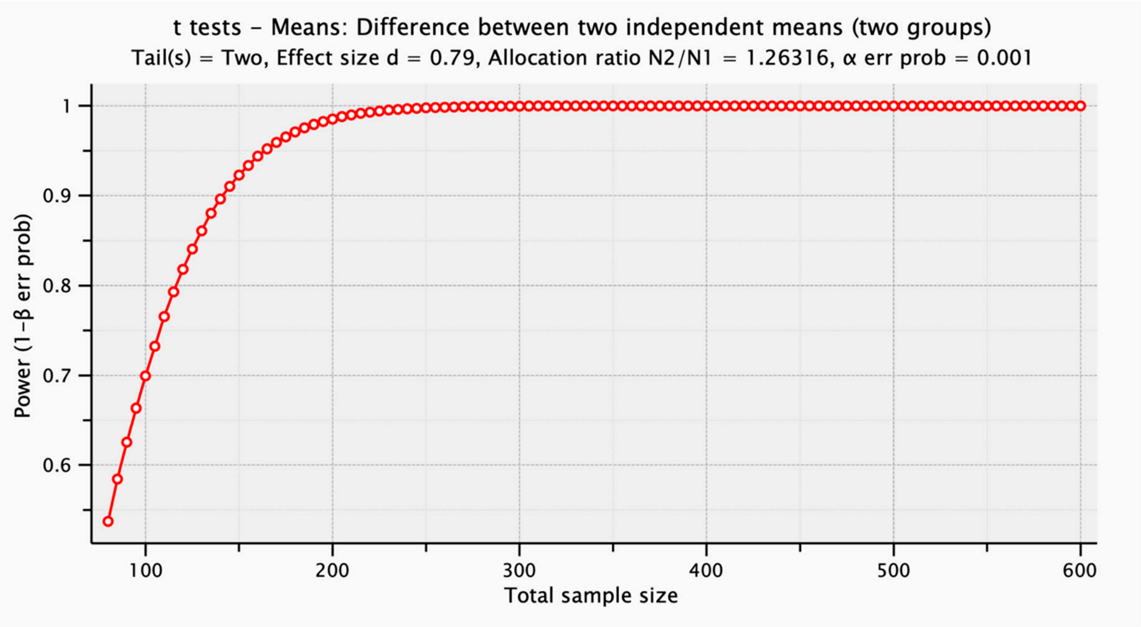

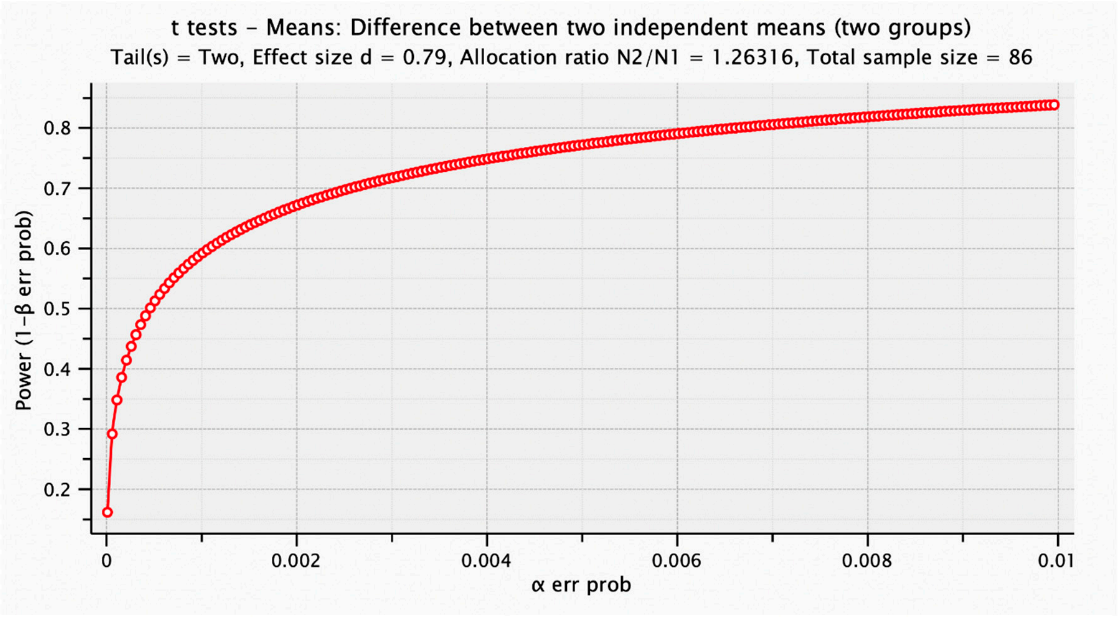

Results

SCRN1 correlation with CSF biomarkers

The matrix below shows the spearman correlations between SCRN1 NPX values (in the plots, this is NPX) and the CSF biomarkers. The correlations are estimated by Row-wise method.

Figure s1. (a) spearman's correlation values and (b) scatterplot of SCRN1 and CSF biomarkers. (c) Scatter plot of SCRN1 NPX values and $\log_2$ of p-tau181 (spearman's rho = 0.8, p-value <0.0001).

(a)

	NPX	p-tau	tau	AB42	AB40	AB42/AB40	NFL
**NPX**	1.0000	0.6358	0.6073	0.0270	0.4732	-0.4291	0.4265
**p-tau**	0.6358	1.0000	0.8719	-0.2904	0.3838	-0.6805	0.5305
**Tau**	0.6073	0.8719	1.0000	-0.2082	0.3842	-0.5849	0.4789
**AB42**	0.0270	-0.2904	-0.2082	1.0000	0.6219	0.7022	-0.0893
**AB40**	0.4732	0.3838	0.3842	0.6219	1.0000	-0.0700	0.3397
**AB42/AB40**	-0.4291	-0.6805	-0.5849	0.7022	-0.0700	1.0000	-0.3522
**NFL**	0.4265	0.5305	0.4789	-0.0893	0.3397	-0.3522	1.0000

(b)

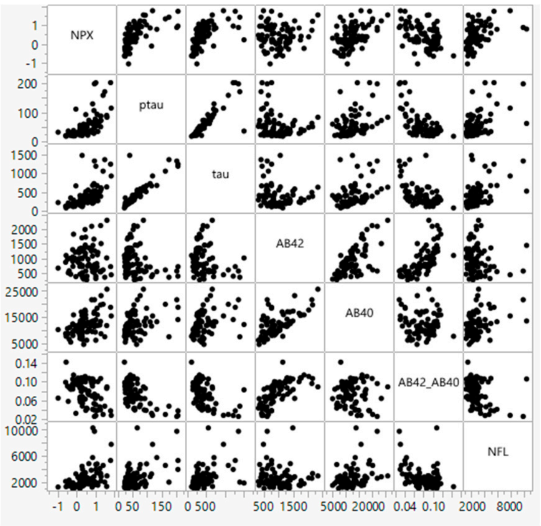

(c)

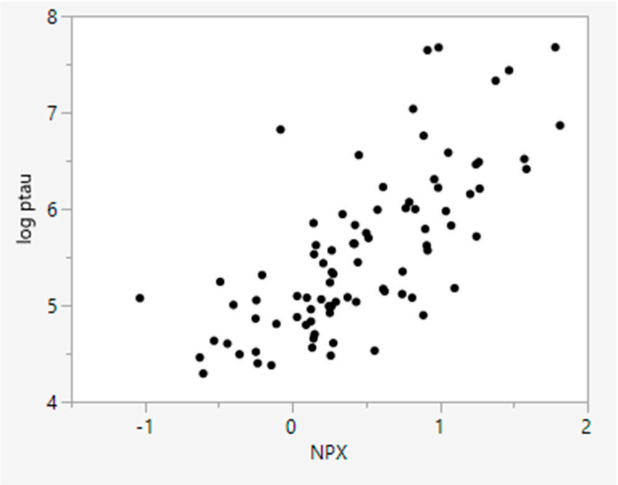

Supplement: Supplementary file 1 [file biomolecules-13-01094-s001.zip › Supplementary Material File S1.pdf]
